# Perfectionism, obsessive-compulsory behaviour, and anxiety in young adults: a moderated mediation model of mobile phone addiction

**DOI:** 10.1186/s12889-025-24119-8

**Published:** 2025-08-19

**Authors:** Jiao Cheng, Yanjun Chen, Jin Liu, Sirui Gao, Yumeng Ju, Bangshan Liu, Zhengzong Liu, Yan Zhang

**Affiliations:** 1 The Second Xiangya Hospital, The Chinese Communist Youth League, Central South University, Changsha, 410011 Hunan China; 2 Department of Psychiatry, and National Clinical Research Center for Mental Disorders, National Center for Mental Disorders, The Second Xiangya Hospital of Central South University, Changsha, 410011 Hunan China; 3 The Chinese Communist Youth League, Central South University, Changsha, 410011 Hunan China

**Keywords:** Moderated mediation, Mobile phone addiction, Perfectionism, Obsessive-compulsory behaviour, Anxiety

## Abstract

**Background:**

Mobile phone addiction is becoming a topical concern among young adults. Recent research argues that mobile phone addiction is related to perfectionism, anxiety, and obsessive-compulsory behaviour. Yet, there is a lack of an integrated model encompassing these factors to explain mobile phone addiction. Guided by a moderated mediation framework, the study aimed to investigate whether perfectionism predisposes young adults to mobile phone addiction through obsessive-compulsory behaviour and anxiety.

**Methods:**

A cross-sectional survey was used to examine all the variables among 1404 Chinese universities’ student.

**Results:**

The results a prevalence of 56.50% in mobile phone addiction among the participants. The direct association between perfectionism and mobile phone addiction was significant (β = 0.12, *95%CI [0.09*,* 0.15]*). Obsessive-compulsory behaviour partially mediated the relationship between perfectionism and mobile phone addiction (indirect effect = 0.08, *95%CI [0.06*,* 0.10]*). Additionally, as anxiety increased, the association between perfectionism and mobile phone addiction strengthened via obsessive-compulsory behaviour (Index = 0.008, *95%CI [0.001*,* 0.004]*).

**Conclusions:**

This large-scale study revealed that individuals high in perfectionism and anxiety are vulnerable for mobile phone addiction, with compulsive use of mobile phone serving as a maladaptive coping strategy to manage distress. The findings contribute to an integrated model that incorporates personality traits, cognitive-behavioral processes, and emotional factors in the development of mobile phone addiction. Perfectionism-informed education and intervention is encouraged to prevent mobile phone addiction in education, clinical, and public health instances.

## Introduction

Mobile phone has become an indispensable tool in individuals’ routines, entertainment, study, and work. Mobile phone addiction is typically regarded as a behavioural addiction that is characterized by cravings for accessing to mobile phone, loss of control over usage, and significant dysfunction in life due to excessive use of mobile phone [1–3]. In the fifth edition of the Diagnostic and Statistical Manual (DSM-5), Internet Addiction was included as a specific type of psychiatric condition, emphasizing its clinical significance [4]. A recent meta-analysis has shown that 1/4 of the general population across the globe is affected by digital addiction, with the burden being significantly higher in low- and middle-income countries [5]. Young adults, especially university students, are the primary users of mobile phones in China, where the prevalence of mobile phone addiction has reached nearly one-third [6]. Mobile phone addiction is detrimental to young adults’ mental health, interpersonal relationships, academic performance, and career development [5–9]. Hence, it is critical to identify the risk factors contributing to young adults’ mobile phone addiction and develop targeted early intervention strategies.

### Perfectionism and mobile phone addiction

One of the similar constructs of mobile phone addiction is problematic mobile phone use, characterized by excessive phone use that interferes with daily functioning, well-being, or interpersonal relationships [1, 10]. Yet, the pathological nature of the symptoms of mobile phone addiction extends beyond excessive use of mobile phone. Some scholars propose that mobile phone addiction can be seemed as the severe end of the continuum of problematic mobile phone use [1, 3]. According to Brown’s behavioural addiction framework, the major characteristics of behavioural addiction includes cognitive-behavioural salience (dominating power of an activity in one’s thinking and life), euphoria (overt pleasure resulting from engaging in the activity), withdrawal (suffer from great psychological distress when the activity is unavailable), dysfunction in life, loss of control, and relapse (a difficulty in reducing attempts for the activity) [11, 12]. Previous research found that many of the mobile phone use behaviour among the young adults fulfilled the criteria for behavioural addiction, for instance, experiencing distress when mobile phone was not available [13]. Further, functional impairment, for instance, cognitive failures, is also prevalent in young adults with mobile phone addiction, suggesting the clinically significant symptomology of mobile phone addiction [14, 15].

There have been different explanatory models for mobile phone addiction. Nevertheless, research generally believes that behavioural addiction, in order words, an over-dependence on an activity, is rooted in psychological factors [16]. In recent years, research has burgeoned on the association between personality and mobile phone addiction. Pathological personality was found related to the development of mobile phone addiction [17, 18]. A meta-analysis evident that higher neuroticism, characterized by an increased anxiety and distress, and lower conscientiousness, characterized by an reduced sense of responsibility and efficiency, predicted inclination to mobile phone addiction according to the Big Five personality model [19]. Research also suggests that type D personality, characterized by a tendency to feel negative emotions and a discomfort in social interactions and, was associated with addictive behaviour related to social media use [20, 21].

Perfectionism as a personality trait is widely found to be related to the development of mobile phone addiction as a transdiagnostic risk factor [8, 22, 23]. Individuals with perfectionism tend to set exceedingly high standards for themselves and is intolerant of mistakes and imperfection [24]. While adaptive perfectionism can foster self-development, it can become maladaptive when the individual is intolerant of making mistake and engaging in self-criticism, giving rise to psychological distress and a range of psychopathologies such as anxiety and addictive behaviour [24–27]. Research showed that individuals with maladaptive perfectionism may be predisposed to mobile phone addiction as an result of the anxiety and frustration after failures and mistake [8, 22, 23]. It is argued that individuals with maladaptive perfectionism is predisposed to dependency on mobile phones as a coping strategies for the anxiety derived from the gap between expectation and reality [22]. Nevertheless, it remains underdeveloped the mechanism between perfectionism and mobile phone addiction.

### The role of obsessive-compulsory behaviour and anxiety

Obsessive-compulsory behaviour has a strong link to perfectionism and mobile phone addiction [27–30]. Obsessive-compulsory behaviour refer to the occurrence of obsessive thoughts and repetitive behaviors even without the presence of pleasure, such as repetitive checking [31]. A study assessing the clinical treatment outcomes of obsessive-compulsory disorder (OCD) provides an empirical evidence of perfectionism as an important maintaining element in OCD [32]. Perfectionism is also considered the dispositional antecedent of OCD [33]. In terms of mobile phone addiction, research has demonstrated that individual who is predisposed to OCD shows more addictive use of mobile phones due to a fear of missing out information and experiences [34, 35]. Therefore, given the existing evidence on the interrelated relationship between obsessive-compulsory behaviour, perfectionism, and mobile phone addiction, it is hypothesized that the obsessive-compulsory behaviour may intervene the association between perfectionism and mobile phone addiction as a mediator.

In addition, it is worth noting the role of anxiety in perfectionism and obsessive-compulsory behaviour. One of the reasons that individuals with perfectionism may experience great anxiety is an intolerance of uncertainty, which may drive a range of pathological behaviours including obsessive-compulsory behaviour [31, 36, 37]. Let al.one the fact that previous research has widely confirmed a positively link between anxiety and mobile phone addiction [7, 10, 38, 39]. Thus, it is further hypothesized that anxiety may serve as a moderator in the association between perfectionism and mobile phone addiction via obsessive-compulsory behaviour.

### Hypothesis

The present study aims to examine a moderated mediation model to investigate whether young adults with perfectionism are predisposed to mobile phone addiction with obsessive-compulsory behaviour serving as a mediator and anxiety serving as a moderator. Based on the existing literature, the following hypotheses were proposed:

- H1: Perfectionism will have a positive impact on mobile phone addiction.
- H2: Obsessive-compulsory behaviour will positively mediate the association between perfectionism and mobile phone addiction.
- H3: Anxiety will moderate the indirect effect of perfectionism on mobile phone addiction through obsessive-compulsive behaviour.

### Moderated mediation model

**Fig. 1:**
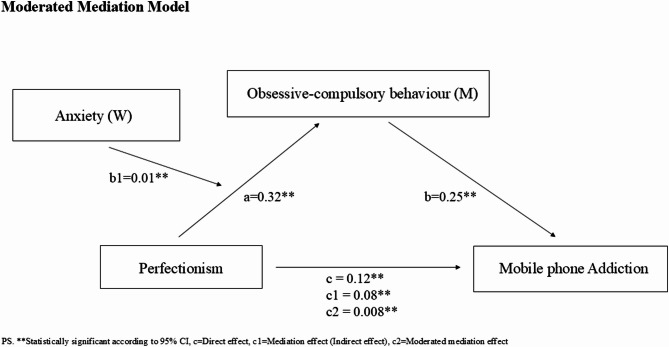
Moderated mediation model

Figure 1 depicts the conceptual moderated mediation model, which examines: (1) Perfectionism to Obsessive-compulsory behaviour (path a), (2) Obsessive-compulsory behaviour to Mobile phone addiction (path b), (3) Perfectionism to Obsessive-compulsory behaviour via Anxiety (path b1), (4) Direct effect: Perfectionism to Mobile phone addiction (path c), (5) Mediation effect: Perfectionism to Mobile phone addiction via Obsessive-compulsory behaviour (path c1), (6) Moderated mediation effect: the interaction (Perfectionism x Anxiety) to Mobile phone addiction via Obsessive-compulsory behaviour (path c2).

## Methods

### Participants and instruments

Chinese universities’ students from various institutions, locations, majors, and socioeconomical background were recruited. The levels of study ranged from first-year undergraduate to post-doctoral degree, with various age groups. A total of 1406 students completed the study, and 1404 valid responses were included for analyses after eliminating 2 extreme and incomplete answers (effective rate = 99.86%). The demographic characteristics of the participants are detailed in the Table 1.

**Table 1:** Characteristics of demographic, total scores of the key variables, and group differences (*N* = 1404)

	Percentages(%)/M ± SD	t/χ2
Demographics/M ± SD		
Gender		
Female	58.80%	43.81**
Male	41.20%	
Age	20.22 ± 8.00	
Place of living		
Cities	47.60%	
Suburban	30.20%	
Countryside	22.20%	
Educational level		
Undergraduate	88.60%	
Masters	10.80%	
Doctors and post-doctor	0.60%	
Mobile phone addiction	30.11 ± 11.95	
Independent	43.50%	353.00**
Dependent	56.50%	
Perfectionism	74.86 ± 25.27	
Obsessive-compulsory behaviour	20.03 ± 15.65	
Anxiety	3.88 ± 4.68	

PS. *p＜0.05,**p＜0.001

A designed online survey was sent out to the students for the purpose of data collection from August 2023 to October 2023 through a widely-used Chinese online survey platform (Sojump), recording the demographic information (i.e., age, gender, educational levels) and the characteristics mobile phone usage, obsessive-compulsory behaviour, and the emotional status (i.e., anxiety) of the participants. The authors pre-tested the survey to ensure the reliability, and the validity of the data collected. There were no material or monetary rewards for participating in the survey. Only the authors had access to the online survey platform and the anonymous responses. Five measures were used in the survey:

#### Mobile phone addiction

The Chinese version *of Mobile Phone Involvement Questionnaire (MPIQ)* was used [2]. MPIQ quantifies cognitive and behavioral features of mobile phone addiction, such as withdrawal, salience, loss of control, relapse, interpersonal conflicts. The MPIQ is an 8-item measure scored on a 7-point Likert scale, ranging from 1 (strongly disagree) to 7 (strongly agree). Example items: “I lose track of how much I am using my mobile phone”. A higher score demonstrates a greater mobile phone addiction. MPIQ has good validity and reliability among Chinese samples, Cronbach’s α = 0.84 [40]. The Chinese version of MPIQ considers a score of over 32 as mobile phone addiction (the sensitivity and the specificity of the MPIQ at this score were 0.634 and 0.652, respectively) [40].

#### Perfectionism

The Chinese version of *Frost’s Multidimensional Perfectionism Scale (FMPS)* was adopted [24]. The FMPS is a self-report measure examining 5 main dimensions of perfectionism covering cognition, emotional, and behavior: concern over mistakes, doubts about actions, parental expectations, high personal standards, and organization. In this study, the 27-items Chinese version of FMPS adopted from was used as it was tested to be valid and reliable among Chinese samples, Cronbach’s α = 0.81 [41, 42]. It is scored on a 5-point Likert scale, ranging from 1 (strongly disagree) to 5 (strongly agree). Example items: “Organization is very important to me”. A higher score demonstrates a greater degree of perfectionism.

#### Obsessive-compulsory behavior

The Chinese version of the *Obsessive–Compulsive Inventory - Revised (OCI-R)* was used [43]. This 18-items measure examined obsessive-compulsory behaviour and the relevant distress in the past month, covering 6 dimensions of obsessive-compulsory behaviour: washing, checking, obsessions, neutralizing, ordering, and hoarding. It is scored on a 5-point scale, ranging from (not at all) to 4 (extremely). OCI-R has good validity and reliability among Chinese samples [44].

#### Anxiety

Anxiety was measured by the *Generalized Anxiety Disorder-7 (GAD-7)* [45]. GAD is a 7-items measure scored on a 3-point scale, ranging from 0 (not at all) to 3 (almost everyday). Scores of 0–4 are normal, 5–9 are mild anxiety, 10–18 are moderate to high anxiety, >19 is severe anxiety. The Chinese version of GAD-7 has good validity and reliability among Chinese samples, Cronbach’s α = 0.91 [46].

### Statistical analysis

The data for the study was exported to Microsoft Excel from the online questionnaire platform anonymously and analyzed by SPSS version 25.0. The significance level was set at *P* < 0.05 (two-tailed). The continuous variables were presented as the mean ± standard deviation (SD). The categorical variables were coded and presented as percentages. Firstly, descriptive data and group comparisons were generated. Secondly, correlations between perfectionism, obsessive-compulsory behaviour, anxiety, and mobile phone addiction (total scores) were examined through Pearson’s correlational analysis. Thirdly, the moderated mediation analysis was performed through the Model 7 of PROCESS version 4.2 in SPSS (bootstrap sample size was set at 5,000) [47]. In the model, the four variables and their abbreviations were defined as: Perfectionism (independent variable, IV); mobile phone addiction (dependent variable, DV); Obsessive-compulsory behaviour (mediator, M); Anxiety (moderator, W).

### Ethical statement

The study was approved by the Ethics Committee of the Second Xiangya Hospital of Central South University (reference: LYF20240210).

## Results

### Demographic information and the total scores of the key variables

Table 1 presented the demographic information of the participants and the total scores of the key variables (*N* = 1404). There were 41.2% male and 58.80% female participants, with a mean age of 20.22 ± 8.00 years old. There were significantly greater female participants in this study (*χ2 = 43.81*,*p*<0.001). A majority of the participants were undergraduates (88.50%) mainly from the cities (47.6%) and suburban (30.20%). The total scores of *MPIQ*,* FMPS*,* OCI-R*, and *GAD-7* were 30.11 ± 11.95, 74.86 ± 25.27, 20.03 ± 15.65, 3.88 ± 4.68, respectively. There were significantly more mobile phone addictive students (56.50%) than non-addictive students (43.50%) (*χ*^2^ = 353.00, *p*<0.001).

### Correlational analysis

There were positive correlational relationships between the key variables (Table 2). Perfectionism was positively correlated with obsessive-compulsory behaviour (*r* = 0.65, *p* < 0.001), anxiety (*r* = 0.34, *p* < 0.001), mobile phone addiction (*r* = 0.47, *p* < 0.001). Mobile phone addiction was positively correlated with obsessive-compulsory behaviour (*r* = 0.49, *p* < 0.001) and anxiety (*r* = 0.32, *p* < 0.001). obsessive-compulsory behaviour was positively correlated with anxiety (*r* = 0.54, *p* < 0.001).

**Table 2:** The correlation matrix of the key variables

	1	2	3	4
1. Perfectionism	1			
2. Obsessive-compulsory behaviour	0.65**	1		
3. Anxiety	0.34**	0.54**	1	
4. Mobile phone addiction	0.47**	0.49**	0.32**	1

PS. **p<0.001

### Moderated mediation model

The moderated mediation analysis (Table 3; Fig. 1) showed that the direct effect (path c) of perfectionism on mobile phone addiction is positive and significant (β = 0.12, *95%CI [0.09*,* 0.15]*), H1 is supported. Secondly, the indirect effect or the mediating effect (path c1) of obsessive-compulsory behaviour in the association between perfectionism and mobile phone addiction was significant (indirect effect = 0.08, *95%CI [0.06*,* 0.10]*), H2 is supported. However, the direct effect of perfectionism on mobile phone addiction was still significant under the mediating effect of obsessive-compulsory behaviour, suggesting that the mediation effect was partial. The mediation effect explained 40% (0.08/0.20) of the total variance. Thirdly, anxiety moderated the mediation effect of obsessive-compulsory behaviour on the association between perfectionism and mobile phone addiction (path c2) and the moderated mediation model was positive and significant (Index = 0.003, *95%CI [0.001*,* 0.004]*), H3 is supported.

**Table 3:** Test of the moderated mediation

	Model 1 (DV = Obsessive-compulsory behaviour)			Model 2 (DV = Mobile phone addiction)		
	Standardized β/Effect/Index	*p*	95%CI	Standardized β	*p*	95%CI
IV = Perfectionism	0.32	*p* <0.001	[0.38, 0.43]	0.12	*p* <0.001	[0.09, 0.15]
M: Obsessive-compulsory behaviour				0.25	*p* <0.001	[0.21, 0.30]
W: Anxiety	1.01	*p* <0.001	[0.97, 1.23]			
IV x W interaction	0.01	*p* <0.001	[0.007, 0.014]			
R^2^	0.54			0.53		
F	557.02**			272.91**		
Direct effect (IV→DV)	0.12	*p* <0.001	[0.09, 0.15]			
Mediation effect (IV→M→DV)	0.08		[0.06, 0.10]			
Moderated mediation Index (IV x W →M→DV)	0.003		[0.001, 0.004]			

*IV*Independent variable, *DV*Dependent variable, *M*Mediator, *W*Moderator

PS. **p<0.001

**Fig. 2:**
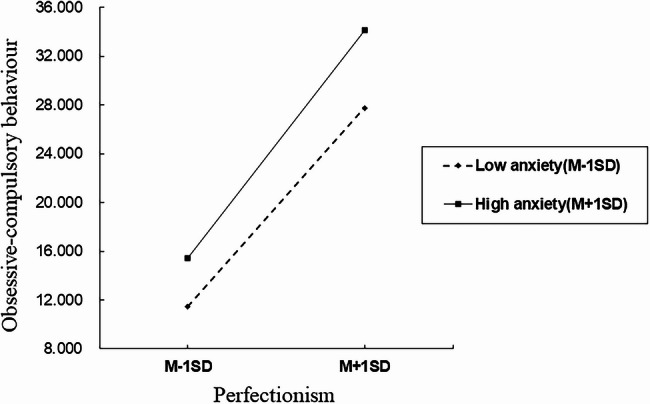
The impact of perfectionism on obsessive-compulsory behaviour under different levels of anxiety

In the model, perfectionism had a significant effect on obsessive-compulsory behaviour (path a) (β = 0.32, *95%CI [0.38*,* 0.43]*). Obsessive-compulsory behaviour has a significant effect on mobile phone addiction (path b) (β = 0.25, *95%CI [0.21*,* 0.30]*). Further, the interaction effect of perfectionism and anxiety on obsessive-compulsory behaviour was positive and significant (path b1) (β = 0.01, *95%CI [0.007*,* 0.014]*). To facilitate the interpretation of the moderating effect of anxiety, a simple slope analysis was used to demonstrate the effect of perfectionism on obsessive-compulsory behaviour under different levels of anxiety (Table 4; Fig. 2): The levels of anxiety were divided into high (*M + 1SD*) and low (*M-1SD*) based on the mean score (M indicating no moderation effect). Results showed that perfectionism had a positive effect on obsessive-compulsory behaviour under both high and low levels of anxiety (*M + 1SD*: β = 0.37, *95%CI [0.34*,* 0.40]*,* M-1SD*: β = 0.28, *95%CI [0.25*,* 0.31]*), with greater effect under high level of anxiety. Similarly, anxiety significantly moderated the overall mediation model (Table 5): Perfectionism had a positive effect on mobile phone addiction via obsessive-compulsory behaviour under both high and low levels of anxiety (*M + 1SD*: β = 0.09, *95%CI* [0.07, 0.11], *M-1SD*: β = 0.07, *95%CI [0.05*,* 0.09]*), with greater effect under high level of anxiety.

**Table 4:** The impact of perfectionism on obsessive-compulsory behaviour under different levels of anxiety

Anxiety	Effect	BootLLCI ~ BootULCI
M (no moderation effect)	0.32	[0.30, 0.35]
M-SD	0.28	[0.25, 0.31]
M + SD	0.37	[0.34, 0.40]

PS. *LL* lower limit, *UL* upper limit

**Table 5:** The impact of perfectionism on mobile phone addiction under different levels of anxiety

Anxiety	Effect	BootLLCI ~ BootULCI
M (no moderation effect)	0.08	[0.06, 0.10]
M-SD	0.07	[0.05, 0.09]
M + SD	0.09	[0.07, 0.11]

PS. *LL* lower limit, *UL* upper limit

## Discussion

The study adopted a population of university student, a generation to have grown up with digital technology, thus, offered a representative cohort to study mobile phone addiction. The results revealed that mobile phone addiction was prevalent in over half of the participants, suggesting that it has become a critical issue among young adults. The findings demonstrated a direct effect of perfectionism on mobile phone addiction, as well as an indirect effect, in which the relationship between perfectionism and mobile phone addiction was positively mediated by obsessive-compulsive behavior. The indirect effect is moderated by anxiety, such that higher anxiety strengthens the indirect effect. These findings support an integrated model that incorporates personality traits, cognitive-behavioral processes, and emotional factors in the development of mobile phone addiction.

### Discussion of the findings

The key finding of the study supports the previous research in which there was a positive direct effect of perfectionism on mobile phone addiction [8, 22, 23]. The current study also complemented previous research in terms of the role of obsessive-compulsory behaviour in mobile phone addiction [29]. It argues that obsessive-compulsory behaviour as a result of perfectionism can strengthen the impact of perfectionism on the development mobile phone addiction. It is a step closer to the mechanism in which perfectionism is related to mobile phone addiction. Although, the partial mediation effect here suggests that potentially more factors need to be taken into consideration in the association between perfectionism and mobile phone addiction association. Moreover, as anxiety increased, both the impact of perfectionism and obsessive-compulsory behaviour on mobile phone addiction was strengthened. This is in line with the findings from previous studies where anxiety seemed to worsen mobile phone addition [7, 38, 39]. The current study argues that anxiety can amplify the impact of obsessive-compulsory behaviour on mobile phone addiction through interacting with perfectionism.

There are some theoretical explanations to the current findings. The first explanatory model is Intolerance of Uncertainty. Intolerance of uncertainty (IU) is characterized by an overestimate and an intolerance of negative outcomes [31, 36, 37]. Existing literature has identified the importance of a sense of control and certainty for individuals with maladaptive perfectionism [31, 48]. IU drives reassurance-seeking, checking behaviors, or avoidance as maladaptive efforts to gain certainty [31, 49]. Anxiety intensifies reactions to uncertainty, thus, individuals with high anxiety and IU are predisposed to engage in obsessive-compulsive behaviors, including overusing mobile phone to regulate distress [49, 50]. Therefore, individuals with perfectionism who experience high anxiety can be particularly vulnerable to obsessive-compulsive behaviors, which ultimately result in mobile phone addiction.

A second explanatory model is the Vulnerability-Stress Model. This model was initially used to explain affective disorders such as depression and schizophrenia. It argued that individuals’ vulnerable factors triggered maladaptive cognitive-behavioral manifestation under psychological or environmental stress (e.g., anxiety)[51, 52]. Recent literature generally supports perfectionism as a trait-based vulnerability to psychopathologies and mobile phone addiction [23, 53, 54]. Anxiety is also widely viewed as a stress-related state, giving rise to maladaptive coping strategies [10, 55]. Thus, the Vulnerability-Stress Model suggests that perfectionism as a trait-based vulnerability can motivate obsessive-compulsive use of mobile phone under high levels of anxiety as a maladaptive coping outcome, which develops into an addiction.

Another explanatory model is the Self-Regulation Failure Theory. Self-regulation refers to the process by which individuals manage and adjust their thoughts, emotions, behaviors, and impulses to cope with challenges and achieve long-term goals [56, 57]. Self-regulation is a type of psychological resources, it can become exhausted when being exploited. Heavy cognition load can exploit self-regulatory resources and cause self-regulation failure [56]. When self-regulation mechanism fails, individual may experience compulsions and addictions [56, 57]. Previous research has revealed that maladaptive perfectionism and anxiety can cause heavy cognition load and exploit self-regulatory resources [58, 59]. As a result of poor self-regulation due to perfectionism and anxiety, individual may have a difficulty in regulating their impulses and behaviours and have to turn to mobile phone to compensate the imbalanced state [14, 60, 61]. As a result, individual fails to regulate their obsessive-compulsory mobile phone use, further contributing to the addictive behavior.

To summarize, the current study has provided empirical evidence to enrich the theoretical framework of mobile phone addiction in young adults, serving as a foundation for future research and relevant intervention strategies.

### Limitations

Limitations of the study relate to the study design and the nature of the data. Firstly, cross-sectional study precludes any claims about the causal effects. Longitudinal designs should be employed in the future to determine the directions of the relationships in the model. Nevertheless, large cross-sectional data is still valuable in terms of identifying risk factors for future studies. Secondly, self-report data may be insufficient to generate external validation of the reported variables. Objective measures of mobile phone use will generate more reliable associations in the future. Thirdly, significant results may have been influenced by potential confounding variables which were not included in the analysis but may have an impact on the variables, such as gender, educational level, state of mind at the time. Gender may be a key confounder considering that the female students significantly outnumbered the male students in this study. Finally, there are many dimensions to mobile phone addiction, such as use maintenance and initiation [10]. It is very likely that the mechanism of each process varies from each other. The measure of *MPIQ* used in this study mainly concerned use maintenance and excessive use of mobile phones [2]. Therefore, the study only explains one of many facets of mobile phone addition, the findings may not be generalized to, for instance, the motivation of using mobile phone.

### Implications

The study offers a ‘personality-emotion-behaviour’ model in explain mobile phone addiction, which inspire its detection, prevention, and intervention. The current study highlighted the direct impact of perfectionism on the development of mobile phone addiction, as well as the indirect impact of obsessive-compulsory behaviour and anxiety. The findings implied that targeted intervention should be provided to young adults with maladaptive perfectionism who are also experiencing obsessive-compulsory behaviour and anxiety, as these individuals may be particularly vulnerable to mobile phone addiction. In the first place, actions from universities are warranted to mitigate mobile phone addiction among the students. Firstly, tutors and student support office should pay extra attention to the students who are particularly perfectionist and easy to self-blame for mistakes. Secondly, perfectionism-informed education for individuals with maladaptive perfectionism is encouraged.It is preferable for universities to collaborate with experts and organizations to facilitate regular psychoeducational workshops targeting maladaptive perfectionism and ways to restore emotional and behavioral health. Previous research suggested that mindfulness-based exercises and self-compassion exercises were acceptable and feasible ways to mitigate maladaptive perfectionism and improve emotion regulation as well as academic performance among university students, given sufficient engagement [62, 63]. In the second place, in terms of clinical contexts, it is evident that Explanatory Feedback Intervention (EFI) is effective and applicable among university students with maladaptive perfectionism which features the management of maladaptive cognitive and behavioural patterns and the facilitation of anxiety and coping strategies [26, 64, 65]. Finally, from a public health perspective, establishing high-risk personality profiles for the population of young adults may benefit strategy and policy-making concerning mobile phone addiction. With respect to future studies, as stated, prospective research is preferred to test this model in a longitudinal design. Future studies are also encouraged to examine this model in a wider cultural background. Additionally, considering the mediation effect of obsessive-compulsory behaviour is only partial, there may be more factors mediating the effect of perfectionism on mobile phone addiction. Future studies may explore this aspect.

In conclusion, this study highlights the significant role of perfectionism in the development of mobile phone addiction among young adults. By adopting a moderated mediation framework, the findings demonstrated that perfectionism positively predicted mobile phone addiction, and this relationship is intensified by higher levels of obsessive-compulsive behavior and anxiety. These results imply a need for institutional interventions targeting perfectionistic tendencies, anxiety, and compulsive behaviors to mitigate the risk for mobile phone addiction in young adults.

## Data Availability

The datasets used and/or analysed during the current study are available from the corresponding author on reasonable request.
